# The New Era of Therapeutic Strategies for the Treatment of Retinitis Pigmentosa: A Narrative Review of Pathomolecular Mechanisms for the Development of Cell-Based Therapies

**DOI:** 10.3390/biomedicines11102656

**Published:** 2023-09-28

**Authors:** Valentina Becherucci, Giacomo Maria Bacci, Elisa Marziali, Andrea Sodi, Franco Bambi, Roberto Caputo

**Affiliations:** 1 Cell Factory Meyer, Children’s Hospital A. Meyer Istituti di Ricovero e Cura a Carattere Scientifico (IRCCS), University of Florence, 50139 Florence, Italy; valentina.becherucci@meyer.it (V.B.); franco.bambi@meyer.it (F.B.); 2 Pediatric Ophthalmology Unit, Children’s Hospital A. Meyer Istituti di Ricovero e Cura a Carattere Scientifico (IRCCS), University of Florence, 50139 Florence, Italy; elisa.marziali@meyer.it (E.M.); roberto.caputo@meyer.it (R.C.); 3 Department of Neuroscience, Psychology, Drug Research and Child Health, University of Florence, 50139 Florence, Italy; andreasodi2@gmail.com

**Keywords:** inherited retinal dystrophies, retinitis pigmentosa, therapeutic strategies, cell therapies

## Abstract

Retinitis pigmentosa, defined more properly as cone–rod dystrophy, is a paradigm of inherited diffuse retinal dystrophies, one of the rare diseases with the highest prevalence in the worldwide population and one of the main causes of low vision in the pediatric and elderly age groups. Advancements in and the understanding of molecular biology and gene-editing technologies have raised interest in laying the foundation for new therapeutic strategies for rare diseases. As a consequence, new possibilities for clinicians and patients are arising due to the feasibility of treating such a devastating disorder, reducing its complications. The scope of this review focuses on the pathomolecular mechanisms underlying RP better to understand the prospects of its treatment using innovative approaches.

## 1. Introduction

**Figure 1:**
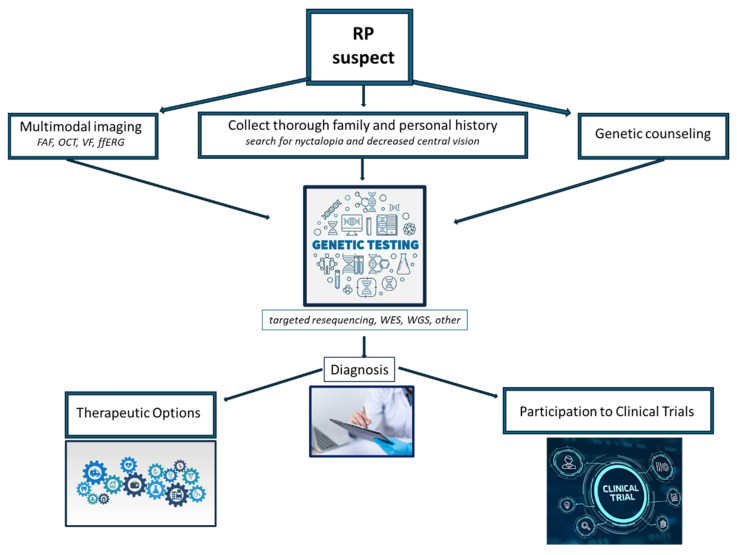
Simplified clinical flow chart for assessing suspected RP. FAF: fundus autofluorescence, OCT: optical coherence tomography, VF: visual field, ffERG: full-field electroretinography, WES: whole-exome sequencing, WGS: whole-genome sequencing.

Retinitis pigmentosa (RP) is a relatively common inherited retinal disorder, with an estimated worldwide mean prevalence of 1:4000 people. It consists of progressive retinal degeneration due to the loss of photoreceptors, leading to severe visual impairment in later stages. RP may be inherited in an autosomal-dominant, autosomal-recessive, or X-linked manner. Some digenic and mitochondrial forms have also been described. Actual RP prevalence is related to the geographic location of the reported study and, in consequence of this, it can vary between 1:750 and 1:9000 individuals [1]. Clinically, the fundus examination usually shows diffuse dystrophy of the retinal pigment epithelium (RPE), waxy optic disc pallor, vessel narrowing, and bone spicule pigmentation. However, atypical forms with an unusual clinical picture may be found in clinical practice. Diagnosis may be easy in typical cases, but it may be challenging in atypical presentations, as the disease may present overlapping features with different retinal disorders; a simplified clinical flow chart for suspected RP is provided in Figure 1. Additionally, RP is associated with an increased risk of other ocular complications, such as cataracts and cystoid macular edema (CME), which may cause additional visual disturbances [2,3,4]. The pathophysiology of RP, with such complications that may affect vision, often leads to significant visual impairment at a young age, which can also have an impact on patients’ physical and mental well-being. Researchers have identified mutations in over 100 genes that contribute to developing non-syndromic RP. Syndromic RP is even more complex due to molecular pathways acting in multiple organs. In the past, RP was thought to be untreatable, but recent medical advancements, such as genetic therapies, offer promising possibilities for slowing down or stopping the degeneration of photoreceptors and potentially even restoring some level of visual function [5]. Due to our enhanced comprehension of the cellular mechanisms and genetic factors associated with RP, as well as the eye’s immune-privileged nature, gene therapy has emerged as a highly promising treatment option for RP [6]. Different studies on animal models have shown the potential benefits of gene therapy for RPE65-associated inherited retinal diseases. As a result, human clinical trials for gene therapy have been initiated, aiming to explore the potential benefits of this novel treatment approach [7,8,9,10]. The positive results in terms of both the safety profiles and clinical endpoints in these trials have led to the approval of Voretigene Neparvovec as the first FDA-approved gene therapy for patients with RPE65-associated retinal dystrophies, now commercially available with the name of Luxturna^®^ [11]. In this review, we aim to discuss and highlight the different therapeutic approaches and the current clinical management of RP, focusing on the alterations in the molecular pathways involved in the development of RP.

## 2. Pathomolecular Mechanisms of Retinitis Pigmentosa

Generally speaking, the pathomolecular mechanisms of RP involve primarily genetic mutations that disrupt the normal functioning of the retina and the retinal pigment epithelium [12] through specific and common pathways.

These genes are involved in different cellular processes within the retina, including phototransduction, visual cycle, photoreceptor structure, and the maintenance of retinal integrity. The specific genes and mutations define the precise mechanisms behind RP in individual cases [13]. Nevertheless, since the genes activate different pathways and multiple mechanisms often converge to a more unspecific process, for better clarity we first describe the major genes (summarized in Table 1) and their roles that are most commonly involved in the pathogenesis of RP. The definition of “major” and “minor” genes has to be taken with caution since it is actually population-specific, but our goal is only to give a general overview, so this part is followed by a discussion on the more general processes leading to retinal degeneration.

### 2.1. Major Genes Involved in RP

#### 2.1.1. Rhodopsin (RHO)

Mutations in the *RHO* gene are a common cause of autosomal dominant retinitis pigmentosa (adRP) [14]. Rhodopsin is a protein found in the rod photoreceptor cells of the retina, and it plays a critical role in the phototransduction pathway, which converts light signals into electrical signals that can be interpreted by the brain [15]. The disruption and degeneration of rod photoreceptor cells are caused by a variety of *RHO* gene mutations that damage the structure or function of rhodopsin. Over 150 types of mutations of the *RHO* gene have been described [16,17]. Most cases present point mutations that determine the substitution of an amino acid, with consequent alteration of the protein’s structure or function. In addition to these mutations, others involving abnormalities in the protein’s folding or trafficking have also been described. Mutations in the rhodopsin gene that lead to the development of autosomal dominant forms of retinitis pigmentosa are divided into three different classes [18], distinguished by the dysfunction of rhodopsin and the nature of its accumulation in cell culture. In class 1 mutations, the photopigment remains functional, and its bond with 11-cis-retinal remains intact, while the accumulation of protein in embryonic cell culture happens specifically on the cytoplasmic membrane. Class 2 mutations cause damage to the composition of the photopigment and the buildup of faulty protein in the endoplasmic reticulum. Class 3 encompasses mutations that result in the production of hyperphosphorylated rhodopsin, which exhibits a strong association with arrestin [19]. The consequent rhodopsin–arrestin complex disturbs the structure of the endosomal compartment and impairs endocyte function. All these mutations have a strong impact on rhodopsin function as they can impair the light-sensitive ability of the protein or disrupt its signaling cascade within the photoreceptor cells [20]. These abnormalities can lead to defects in phototransduction, reduced sensitivity to light, and eventually, to the death of rod photoreceptor cells.

#### 2.1.2. Peripherin/RDS (PRPH2)

Mutations in the *PRPH2* gene can cause autosomal dominant RP. The peripherin/*RDS* protein is involved in the structural integrity and function of photoreceptor outer segments. Specifically, PRPH2 is a transmembrane protein that is mainly expressed in rod and cone photoreceptor cells. It plays a crucial role in the structural integrity and organization of the photoreceptor outer segments, which are responsible for capturing and processing light signals [21]. Mutations occurring in the *PRPH2* gene can give rise to diverse structural anomalies in the peripherin 2 protein [22]. These abnormalities can impact crucial aspects, such as protein folding, stability, and interactions with other proteins. The resultant disruption in the peripherin 2 function can hinder the formation of photoreceptor outer segments, consequently compromising the normal functionality of the photoreceptor cells [23]. One common type of mutation in *PRPH2* is the missense mutation, which can affect the protein’s folding, stability, and ability to interact with other proteins in the photoreceptor cells [24]. Another type of common mutation is the frameshift mutation, mainly associated with anomalies in the protein’s structure and function [22]. Frameshift mutations often result in a truncated or non-functional peripherin/*RDS* protein.

Defective peripherin/*RDS* protein can lead to mislocalization [25], aggregation, or degradation of the protein, affecting the integrity and function of the outer segments. This disruption ultimately results in the progressive degeneration of the photoreceptor cells and the characteristic symptoms of retinitis pigmentosa, such as night blindness, peripheral vision loss, and eventually, central vision impairment.

#### 2.1.3. Cyclic Nucleotide-Gated (CNG) Channels

Mutations in genes encoding the CNG channels, such as *CNGA1* and *CNGB1*, are associated with autosomal recessive RP [26]. These channels are located in the outer segment of rod and cone photoreceptor cells, and they are involved in the regulation of ion influx in response to light stimulation. They are responsible for the regulation of intracellular calcium and sodium ions, which are essential for phototransduction (the process by which light signals are converted into electrical signals in the retina thanks to hyperpolarization/depolarization phenomena) [27]. Mutations in genes encoding CNG channels can impair the normal function of these channels, disrupting the phototransduction process and leading to RP [28]. Mutations in the *CNGB1* and *CNGA1* genes, which encode subunits of the CNG channels, have been associated with autosomal recessive RP [29]. Mutations in the *GNAT2* gene, which encodes the transducin alpha-subunit involved in CNG channel regulation, have also been linked to autosomal dominant RP. Impaired CNG channel function leads to abnormalities in the phototransduction process, where the conversion of light stimuli into electrical signals is disrupted [30]. This alteration can result in reduced sensitivity to light, decreased visual acuity, and progressive vision loss, which are characteristic symptoms of RP. Additionally, dysfunctional CNG channels can lead to cellular stress and oxidative damage, and ultimately trigger photoreceptor cell death [31]. The loss of photoreceptor cells further contributes to the degeneration of the retina and the progression of RP.

#### 2.1.4. Retinal Pigment Epithelium-Specific 65 kDa Protein (RPE65)

Mutations in the *RPE65* gene are associated with autosomal recessive RP forms [32]. The *RPE65* gene encodes a protein called retinoid isomerohydrolase, which is primarily expressed in the retinal pigment epithelium (RPE) cells. RPE65 is involved in the visual cycle, a process that regenerates the visual pigment rhodopsin in photoreceptor cells. It plays a crucial role in converting all-trans-retinol to 11-cis-retinal, which is essential for the proper functioning of photoreceptor cells [33]. Mutations in the *RPE65* gene result in a loss or dysfunction of the RPE65 protein, disrupting the visual cycle and impairing the regeneration of 11-cis-retinal [34]. As a consequence, there is a decreased availability of 11-cis-retinal, leading to compromised phototransduction and eventual degeneration of photoreceptor cells [32,35]. Mutations in the *RPE65* gene are also associated with a severe form of retinitis pigmentosa known as Leber congenital amaurosis (LCA) or severe early childhood-onset retinal dystrophy (SECORD).

#### 2.1.5. Retinitis Pigmentosa GTPase Regulator (RPGR)

Mutations in the *RPGR* gene are a major cause of X-linked RP (XLRP), which primarily affects males, as the *RPGR* gene is located on the X chromosome and is involved in the structure and function of the photoreceptor connecting cilium [36,37]. The RPGR protein is indeed predominantly localized in the connecting cilium and outer segment of photoreceptor cells in the retina. These cellular structures play critical roles in the phototransduction cascade and the maintenance of normal vision [38]. Mutations in the *RPGR* gene can affect the normal function of the RPGR protein, leading to retinal degeneration in XLRP [39]. The specific pathogenic mechanisms underlying *RPGR*-related retinal degeneration are not fully understood. However, it is believed that the mutations result in impaired ciliary transport, altered protein–protein interactions, or disrupted signaling pathways, ultimately leading to photoreceptor cell death and vision loss [36,40].

#### 2.1.6. Cone–Rod Homeobox Protein (CRX)

Mutations in the *CRX* gene are associated with the autosomal-dominant RP form [41]. The cone–rod homeobox protein is a transcription factor that plays a crucial role in the development and function of photoreceptor cells in the retina. *CRX* is primarily expressed in the cone and rod photoreceptor cells of the retina, where it regulates the expression of genes involved in photoreceptor development, differentiation, and maintenance [42]. *CRX* is essential for the proper formation and function of these specialized cells, which are responsible for capturing and processing light signals [43]. *CRX* regulates the expression of genes encoding various photoreceptor-specific proteins, including opsins (light-sensitive pigments), transducin, and other important components of the phototransduction pathway. It helps establish the unique characteristics and functions of cone and rod photoreceptor cells, ensuring their proper light-sensitive abilities [44]. Mutations in the *CRX* gene can disrupt the normal function of the CRX protein, leading to impaired development and function of photoreceptor cells [43]. This can result in the degeneration of cones and rods with different specific effects depending on the kind of *CRX* mutation, resulting in isolated cone dysfunction to more generalized cone-rod dystrophy [45].

#### 2.1.7. Usher Syndrome Genes

It has been demonstrated that some forms of RP are associated with Usher syndrome [46], which involves both hearing loss and vision impairment. Genes associated with Usher syndrome, such as *USH2A*, *MYO7A*, *CLRN1*, and *CDH23*, can cause RP, in addition to other symptoms. It is a heterogeneous condition with several genes implicated in its development. The most commonly associated genes with Usher syndrome and RP include the following:

Mutations in the *MYO7A* gene account for the majority of Usher syndrome type 1 (USH1) cases [47]. *MYO7A* encodes the protein myosin VIIA, which is involved in the structure and function of hair cells in the inner ear and the development and maintenance of photoreceptor cells in the retina. It plays an important role in the renewal of the outer photoreceptor discs, in the distribution and migration of retinal pigment epithelium melanosomes and phagosomes, and in the regulation of opsin transport in retinal photoreceptors [48]. Mutations in the *USH1C* gene are also associated with Usher syndrome type 1. The *USH1C* gene encodes harmony, a scaffolding protein involved in the organization of hair cell stereocilia and the synaptic connections in the retina [47].

Mutations in the *USH2A* gene are the most common cause of Usher syndrome type 2 (USH2). The *USH2A* gene encodes usherin, a protein involved in the maintenance of the structure and function of the photoreceptor cells and the hair cells of the inner ear [49].

Mutations in the *GPR98* gene, also known as adhesion G protein-coupled receptor V1 (*ADGRV1*) are associated with Usher syndrome type 2. The *GPR98* gene encodes the protein G protein-coupled receptor 98, which is involved in the development and function of sensory cells in the inner ear and the retina [50].

Mutations in the *CLRN1* gene are associated with Usher syndrome type 3. The *CLRN1* gene encodes clarin-1, a protein found in the hair cells of the inner ear and the photoreceptor cells of the retina [51], which seems to play an important role in the development and homeostasis as a regulatory element for the synapses within the retina.

### 2.2. Mechanisms Involved in RP

As discussed above, the pathogenesis of RP involves a complex interplay of genetic, biochemical [52], and cellular mechanisms that ultimately lead to the degeneration of photoreceptor cells in the retina [53]. Before proceeding, we think that is noteworthy to underline the extreme genetic heterogeneity of RP: the increasing number of papers describing novel mutations and novel genes implicated in the pathogenesis of RP represent evidence of the work that has yet to be carried out to define a proper genotype–phenotype relationship to predict the role of such variants in determining specific isolated or syndromic forms. For instance, a recent paper by Smirnov et al. [54] showed the wide range of presentation of the potentially life-threatening *CLN3* mutations: the authors found that a genotype–phenotype relationship could be extremely useful to predict isolated retinal dystrophy from a devastating early-onset disorder with neurological involvement.

**Figure 2:**
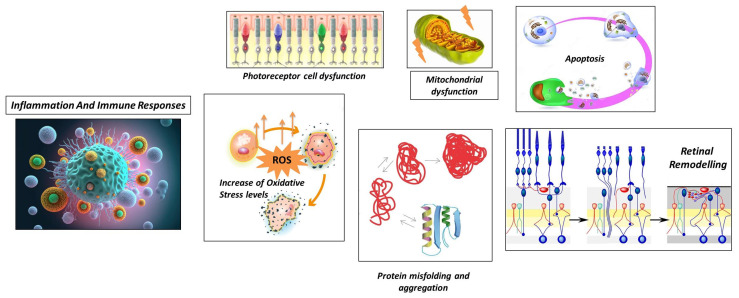
Schematic representation of general processes underlying retinitis pigmentosa.

Although the exact pathogenesis can change depending on the specific genetic mutations that act as initial triggers, in the next section we describe an overview of more general and common processes that contribute to the development and progression of RP, which are also briefly illustrated in Figure 2.

#### 2.2.1. Photoreceptor Cell Dysfunction

The first pathogenetic mechanism is photoreceptor cell dysfunction. RP primarily affects the photoreceptor cells in the retina, where mutations in the genes involved in the visual cycle, phototransduction, and photoreceptor structure can lead to impaired functioning of these cells, affecting the structure and function of photoreceptor proteins [55].

#### 2.2.2. Protein Misfolding and Aggregation

Another mechanism that contributes to the development of retinitis pigmentosa is represented by protein misfolding and aggregation [56]. In this case, mutations in specific genes can lead to the production of misfolded or unstable proteins. Abnormal proteins can accumulate within photoreceptor cells and trigger cellular stress responses, including endoplasmic reticulum stress and unfolded protein response. The accumulation of misfolded proteins and the activation of stress pathways can contribute to photoreceptor cell dysfunction and degeneration [57].

#### 2.2.3. Increase in Oxidative Stress Levels

Another important pathomolecular mechanism is the increase in oxidative stress levels, encouraged by damage to the normal cellular processes in the retina.

The accumulation of reactive oxygen species (ROS) can damage cellular components, including DNA, proteins, and lipids. This oxidative stress can further contribute to the degeneration of photoreceptor cells in RP [58]. In RP, oxidative stress occurs due to multiple factors related to the degenerative processes in the retina. In RP, there is evidence of decreased antioxidant capacity in the retina, which results in an imbalance between ROS production and antioxidant defenses (impaired antioxidant defense) [59]. This imbalance results in the accumulation of ROS, which can damage cellular components, including photoreceptor cells [60]. The consequences of oxidative stress in RP contribute to the ongoing degeneration of photoreceptor cells, exacerbating the visual impairment experienced by individuals with the condition [61]. Strategies aimed at reducing oxidative stress and enhancing the antioxidant defense system have been investigated as potential therapeutic approaches for RP [62]. These include the use of antioxidants, such as vitamins C and E, and other compounds that can mitigate ROS-induced damage [63,64].

#### 2.2.4. Mitochondrial Dysfunction

In the context of retinitis pigmentosa, another important pathogenetic mechanism is mitochondrial dysfunction, as it contributes to the disease’s progression and the degeneration of photoreceptor cells in the retina [65].

Mutations in the genes associated with mitochondrial function, such as those encoding mitochondrial proteins or involved in mitochondrial DNA maintenance, can lead to mitochondrial dysfunction in RP. Impaired energy production and increased oxidative stress associated with dysfunctional mitochondria can contribute to photoreceptor cell death [66]. Mitochondria are responsible for generating energy in the form of adenosine triphosphate (ATP) through oxidative phosphorylation, and due to their role in capturing and processing light signals, photoreceptor cells have high energy demands [67]. Mitochondrial dysfunction in RP can lead to impaired ATP production, resulting in energy deficits that can compromise the survival and function of photoreceptor cells [68]. Moreover, mitochondrial dysfunction can contribute to an imbalance between the production and scavenging of reactive oxygen species, as ROS are byproducts of mitochondrial metabolism, and excessive ROS production due to mitochondrial dysfunction can lead to oxidative stress, causing damage to cellular components, including photoreceptor cells [69]. Mitochondria are also involved in regulating calcium homeostasis in cells. The disruption of calcium signaling due to mitochondrial dysfunction can impact various cellular processes, including phototransduction and cell survival [70]. Dysregulated calcium levels can trigger apoptotic pathways and contribute to the degeneration of photoreceptor cells in RP [71].

Mitochondria play a critical role in apoptosis, and their dysfunction can trigger the release of pro-apoptotic factors, such as cytochrome c, leading to the activation of apoptotic pathways and the subsequent death of photoreceptor cells [65]. Finally, mitochondrial dysfunction can result in the accumulation of toxic reactive metabolites, such as reactive aldehydes and lipid peroxidation products. These metabolites can damage cellular components, including lipids, proteins, and DNA, further contributing to the degeneration of photoreceptor cells in RP [65]. The exact mechanisms underlying mitochondrial dysfunction in RP can vary depending on the specific genetic mutations involved. Several genes associated with RP are known to affect mitochondrial function directly or indirectly [55]. Understanding the specific mitochondrial defects and their impact on retinal cells is essential for developing targeted therapeutic strategies to mitigate mitochondrial dysfunction and potentially slow down the progression of RP [72].

#### 2.2.5. Apoptosis

Apoptosis represents another pathogenetic mechanism underlying RP [73]. Mutations in genes, such as *FASLG*, *FOS*, and *NRL*, have been implicated in regulating apoptosis in photoreceptor cells. The dysregulation of these genes can lead to increased cell death and accelerate the progression of RP [74]. The principal mechanism underlying the activation of the apoptosis process in retinitis pigmentosa is photoreceptor cell stress. As described before, photoreceptor cell stress can arise from factors such as oxidative stress, mitochondrial dysfunction, calcium dysregulation, and protein misfolding underlying molecular defects, which can activate apoptotic pathways [75]. Classic apoptotic pathways are activated by cellular stress in RP, involving the activation of pro-apoptotic proteins and the release of cytochrome c from the mitochondria, finally leading to caspase activation [73]. The progressive loss of photoreceptor cells in the retina occurs as the apoptotic process in RP progresses over time. At first, the rod photoreceptor cells are more severely affected, resulting in night blindness and peripheral vision loss [76]. Later, cone photoreceptor cells may also undergo apoptosis, resulting in further visual impairment, including central vision loss and color vision defects.

#### 2.2.6. Retinal Remodeling

Another remarkable mechanism, characteristic of retinitis pigmentosa, is called retinal remodeling. Retinal remodeling is a phenomenon that refers to the structural and functional changes that take place in the retina in response to the progressive degeneration of photoreceptor cells [77]. These alterations include the synaptic connections and gene expression profiles of various retinal cell types, contributing to the overall dysfunction of the retina. The pathogenetic mechanism underlying retinal remodeling can be classified into four different kinds of alterations, involving different kinds of retinal cells [78]:

1. Neuronal Rearrangement: As photoreceptor cells degenerate, the remaining retinal neurons, including bipolar cells, horizontal cells, and amacrine cells, undergo reorganization. These neurons undergo structural changes and establish new connections with each other to compensate for the loss of photoreceptor input [79].
2. Bipolar Cell Dystrophy: The second-order neurons in the visual pathway, bipolar cells, also undergo structural and functional changes in RP. Abnormal dendritic sprouting or retraction may occur, leading to the formation of ectopic synapses [80]. These changes can result in altered signal processing and contribute to visual abnormalities in RP [81].
3. Müller Cell Gliosis: Müller cells are the major glial cells in the retina and play a crucial role in maintaining retinal homeostasis. In response to photoreceptor cell degeneration, Müller cells undergo gliotic changes, becoming activated and hypertrophic [82]. This gliosis involves changes in gene expression, increased production of glial fibrillary acidic protein (GFAP), and alterations to their structural morphology. Müller cell gliosis can have both protective and detrimental effects on retinal function and can influence the survival and function of the remaining retinal neurons [83].
4. Synaptic Remodeling: Synaptic connections in the retina are reorganized in RP. As photoreceptor cells degenerate, the synaptic connections between photoreceptor cells and downstream neurons, such as bipolar cells and horizontal cells, alter. As a consequence, bipolar cells and surviving cones or bipolar cells and other retinal neurons may form new synaptic connections [84]. This synaptic remodeling can lead to altered signal processing and contribute to the rewiring of the retinal circuitry [85].

Retinal remodeling in RP has severe functional consequences. Rewiring the retinal circuitry can enable surviving neurons to receive input from a wider area of the retina, potentially improving their sensitivity to light. The remodeling processes can also interfere with normal signal processing and cause visual dysfunction, resulting in changes in visual acuity, contrast sensitivity, and color vision [86,87]. Understanding retinal remodeling in RP is important for the development of potential therapeutic interventions. Strategies aimed at modulating or harnessing the adaptive aspects of retinal remodeling while minimizing the maladaptive changes are being investigated as potential approaches to slow down the progression of the disease and restore visual function in RP patients [88].

#### 2.2.7. Inflammation and Immune Responses

Inflammation and immune responses [71] are the final important pathogenetic mechanisms that significantly contribute to the onset and progression of retinitis pigmentosa. While genetic mutations are the primary cause of RP, inflammation and immune responses can exacerbate degenerative processes in the retina. They can be classified into five different mechanisms:

1. Microglial Activation: In response to photoreceptor cell death and degeneration, microglia, the resident immune cells of the retina, become activated. Activated microglia release pro-inflammatory cytokines, chemokines, and reactive oxygen species. While microglial activation initially aims to clear debris and promote tissue repair, chronic or excessive activation can lead to neuroinflammation and further damage to the retina [89,90].
2. Infiltration of Immune Cells: Immune cells from the bloodstream can infiltrate the retina in some cases of RP, which further contributes to the inflammatory response. The release of inflammatory mediators by immune cells, including macrophages and T cells, can worsen retinal damage [91].
3. Cytokine Imbalance: In RP, there is evidence of an imbalance in cytokine signaling in the retina. Pro-inflammatory cytokines, such as tumor necrosis factor-alpha (TNF-α), interleukin-1 beta (IL-1β), and interleukin-6 (IL-6), are upregulated, while anti-inflammatory cytokines, such as interleukin-10 (IL-10) and transforming growth factor-beta (TGF-β), are downregulated. This imbalance can perpetuate the inflammatory response and contribute to the degeneration of photoreceptor cells [92,93].
4. Complement System Activation: Activation of the complement system can lead to the deposition of complement proteins on photoreceptor cells and subsequent immune-mediated damage [91].
5. Oxidative Stress and Inflammation: The imbalance between reactive oxygen species production and antioxidant defense mechanisms can lead to oxidative stress, which can further cause inflammation in RP. The inflammatory cascade and retinal damage can be exacerbated by ROS activating various intracellular signaling pathways involved in inflammatory responses [94].

Modulating immune responses through anti-inflammatory strategies, immunomodulatory therapies, or targeted interventions to regulate specific immune cell functions are areas of active research for potential therapeutic approaches in RP [95].

## 3. Cell-Based Therapies for Retinitis Pigmentosa

Understanding the interplay between inflammation, immune responses, and the degenerative processes in RP is crucial for developing targeted therapies aimed at modulating the immune system, reducing inflammation, and preserving retinal function. The next section will cover the development of cell-based therapies for retinitis pigmentosa and the latest updates.

As previously mentioned, comprehending all the molecular mechanisms that underlie RP is crucial for the development of targeted therapies. The current research effort is focused on strategies, such as gene therapy, stem cell transplantation, neuroprotective agents, and optogenetics, to address specific molecular defects and slow down the progression of RP [52].

**Figure 3:**
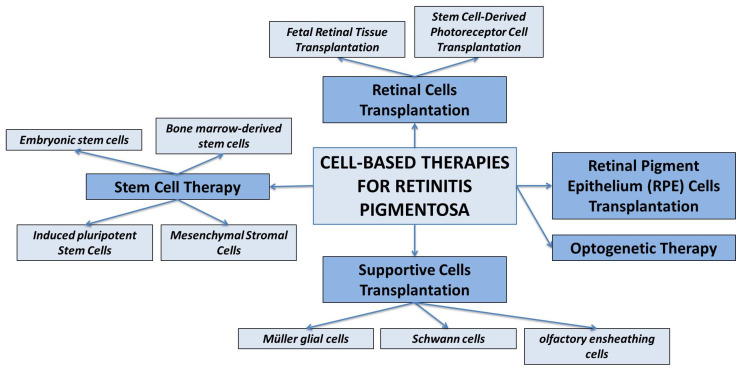
Schematic representation of various therapeutic strategies for treating retinitis pigmentosa.

In this context, particularly cell-based therapies hold great promise for the treatment of RP [96], aiming to replace or restore the function of degenerated photoreceptor cells in the retina or to release some growth factors to enhance the cell survival, growth, and function of retinal cells. The main approaches to cell-based therapy for the treatment of RP are discussed below and summarized in Figure 3.

### 3.1. Retinal Cells Transplantation

This approach involves transplanting healthy retinal cells, such as photoreceptor cells or RPE cells, into the degenerated retina. These transplanted cells can integrate into the existing retinal tissue and potentially restore visual function [97]. Various sources of donor cells are being investigated, including stem cell-derived photoreceptor cells and RPE cells. The goal of photoreceptor cell transplantation is to replace the lost or damaged photoreceptor cells in the retina, and several approaches are being explored, including:

- Fetal Retinal Tissue Transplantation. Fetal retinal tissue, obtained from donor fetuses, can be transplanted into the subretinal space of RP patients. The transplanted cells can integrate into the host retina and potentially improve visual function. However, the availability of fetal tissue is limited, and immunological compatibility needs to be considered [98].
- Stem Cell-Derived Photoreceptor Cell Transplantation. Pluripotent stem cells, such as embryonic stem cells (ESCs) and induced pluripotent stem cells (iPSCs), can be differentiated into photoreceptor-like cells in vitro [99,100]. These cells can then be transplanted into the retina to replace the degenerated photoreceptor cells.

### 3.2. RPE Cell Transplantation

On the other hand, RPE cell transplantation is described as the most innovative and easiest-to-apply therapeutic approach [101]. The RPE plays a critical role in supporting and maintaining the health of photoreceptor cells. It is established that in RP, RPE dysfunction contributes to photoreceptor degeneration. RPE cell transplantation aims to replace the damaged RPE cells and provide a supportive environment for photoreceptor survival. RPE cells can be derived from different sources, and different therapeutic approaches have been described [102].

### 3.3. Supportive Cell Transplantation

In addition to photoreceptor and RPE cells, other supportive cell types can be transplanted to enhance the survival and function of existing retinal cells. For example, Müller glial cells, Schwann cells, central nervous system stem cells, or olfactory ensheathing cells (OECs) can be transplanted to provide neurotrophic support, promote retinal regeneration, and modulate the retinal microenvironment [103,104,105].

### 3.4. Stem Cell Therapy

Stem cell therapy holds significant promise for the treatment of retinitis pigmentosa, as stem cells have the potential to differentiate into various cell types, including photoreceptor cells and RPE cells, aiming to replace the lost or damaged photoreceptor cells and restore visual function [106,107,108]. Several therapeutic approaches have been reported using different kinds of stem cells, like pluripotent stem cells, such as ESCs and iPSCs, and bone marrow-derived stem cells, including multipotent cells, such as MSCs (mesenchymal stromal cells).

ESCs and iPSCs have the remarkable ability to differentiate into various cell types, including retinal cells, like photoreceptors and other retinal neurons, with direct retinal-like cell transplantation. As it is known, RPE cells play a crucial role in supporting and maintaining the health of photoreceptor cells. Dysfunctional RPE cells contribute to photoreceptor degeneration in RP. Pluripotent stem cells can be differentiated into stem cell-derived RPE cells for subretinal space transplantation to restore RPE function. These transplanted cells can provide nutritional support, phagocytosis of the photoreceptor outer segments, and maintain the blood–retinal barrier [109,110]. On the other hand, photoreceptor cell replacement can represent a remarkable therapeutic alternative. In this approach, stem cell-derived photoreceptor cells can be transplanted into the subretinal space of RP patients to replace the lost or damaged photoreceptor cells. Different protocols are used to generate photoreceptor-like cells from pluripotent stem cells, aiming to recapitulate the development and function of native photoreceptor cells. Transplanted photoreceptor cells should integrate into the host retina and establish functional connections with the remaining retinal circuitry [111,112].

A very promising type of adult stem cells in this field are the mesenchymal stromal cells that allow for overcoming the limits represented by the use of pluripotent stem cells, such as ethical conflicts, immune rejection issues, and the risk of viral integrations and oncogene expression. MSCs are a type of adult stem cell that can be obtained from various sources, including bone marrow, adipose tissue, and umbilical cord tissue [113]. The idea behind using MSCs for RP treatment is to harness their regenerative and immunomodulatory properties to protect or replace damaged retinal cells and preserve or restore vision, according to different therapeutic mechanisms, such as the release of paracrine factors and the immunomodulatory effects [114,115]. The paracrine effects of MSC are well described in the literature, as MSCs can secrete various growth factors, cytokines, and neurotrophic factors that create a supportive environment for retinal cell survival and function. The paracrine effects of MSCs may help protect and promote the survival of existing retinal cells, including photoreceptors, and potentially slow down the progression of RP [116,117]. Moreover, MSCs have anti-inflammatory properties, resulting in immunomodulatory effects, which can help modulate immune responses in the retina. Thanks to the release of specific cytokines and growth factors, MSCs suppress the activation and proliferation of immune cells, such as T cells and macrophages, and reduce the production of pro-inflammatory cytokines [118]. By modulating the immune response, MSCs may help alleviate the chronic inflammation associated with RP and create a more favorable environment for retinal cell survival. Moreover, MSCs have been shown to have anti-apoptotic (cell survival-promoting) and anti-oxidative effects [119]. These properties can help protect retinal cells from cell death caused by oxidative stress and apoptosis, which are key features of RP pathogenesis. By reducing oxidative stress and promoting cell survival, MSCs may contribute to the preservation of retinal function in RP [120]. Finally, it has been demonstrated that MSCs have strong angiogenic potential, as they can promote the formation of new blood vessels by enhancing angiogenesis [115,121]. This is a crucial aspect because, in some cases of RP, vascular abnormalities and compromised blood flow in the retina contribute to disease progression. Thanks to their angiogenic potential, MSCs may help improve retinal blood supply and support the survival and function of retinal cells.

Although MSCs show promise for RP therapy, further research is needed to optimize the therapeutic strategies, including determining the optimal route and timing of MSC administration, understanding the mechanisms of action, and addressing concerns, such as cell survival, migration, and long-term effects [116]. Clinical trials are ongoing to assess the safety and efficacy of MSC-based therapies in RP, and their outcomes will provide valuable insights into the potential of MSCs for treating this retinal disorder [114].

Finally, other types of cells that are being studied for cell therapy approaches include olfactory ensheathing cells, a type of glial cell capable of continuous growth and regeneration of olfactory axons into the central nervous system, and human neural progenitors [122]. Approaches with these kinds of cells are aimed at providing an intrinsic continuous supply of neurotrophic factors, reducing the gliotic injury response of Muller cells, and rescuing long-term vision function and associated morphologic substrates by protecting dying host neurons [123].

Although stem cell therapies seem promising, there are still some crucial aspects to consider for their in vivo application. For example, therapy for RP requires careful consideration of immunological factors to ensure cell survival and minimize the risk of immune rejection [106,124]. Autologous transplantation could help minimize immune responses. Immune modulation strategies, such as immunosuppressive medications or genetic engineering of cells to evade immune recognition, are also being explored to enhance graft survival.

In addition, there is still a need for the optimization of transplantation techniques, as the success of stem cell therapy for RP depends on several factors, including the efficient generation of desired cell types, transplantation techniques, and the survival, integration, and functionality of transplanted cells [125]. The current research is indeed moving towards refining the differentiation protocols, optimizing cell delivery methods, and promoting the long-term survival and integration of transplanted cells [126,127].

For all these reasons, stem cell therapy for RP is still in the experimental stage, and clinical trials are ongoing to evaluate its safety and efficacy. The development of standardized protocols, ensuring the long-term functionality and stability of transplanted cells, and addressing ethical considerations are still challenges that need to be addressed [128]. However, stem cell therapy has the potential to restore visual function and halt the progression of RP, giving hope for future treatments.

### 3.5. Optogenetic Therapy

Another kind of absolutely innovative cell-based therapy for RP is optogenetic therapy, whose application is proposed in cases where the photoreceptor cells are severely degenerated [129]. Although it differs from conventional stem cell therapy, in which stem cells are directly transplanted into the body, optogenetic therapy utilizes genetically modified proteins to influence the behavior and function of cells. In this method, light-sensitive proteins are introduced into various retinal cells, such as the remaining retinal ganglion cells or bipolar cells, with the aim of restoring light sensitivity [130]. Subsequently, these altered cells become capable of reacting to light and conveying signals to the brain’s visual processing centers. In summary, optogenetic therapy for RP employs light-sensitive proteins, like microbial opsins (e.g., channelrhodopsin and halorhodopsin), which can be introduced into retinal cells lacking photosensitivity. These proteins are responsive to particular wavelengths of light, enabling them to activate or suppress these cells when exposed to light stimulation [131].

On the other hand, non-photosensitive cells, such as retinal ganglion cells (RGCs) or bipolar cells, are genetically modified to express light-sensitive proteins. This can be achieved by delivering viral vectors carrying the genes encoding the light-sensitive proteins into the cells [131]. The viral vectors integrate the genes into the cell’s genome, enabling the cells to produce light-sensitive proteins. After genetically altering the intended cells to express light-sensitive proteins, they become responsive to light stimulation. To achieve this, specialized goggles or glasses equipped with external light sources are employed to deliver precise light wavelengths to the retina. When the light-sensitive proteins are triggered or inhibited by the light, they initiate electrical signals capable of being transmitted to the brain’s visual processing centers. The objective of optogenetic therapy is to reintroduce light sensitivity to non-photosensitive retinal cells, ultimately aiming to restore visual function in individuals with RP [132]. Although the restored vision may not fully replicate natural vision, it can provide the ability to perceive light and distinguish basic shapes and objects, potentially improving the quality of life for patients with very advanced stages of RP [133]. Optogenetic therapy is currently in its early stages of development and research, with ongoing clinical trials assessing its safety and effectiveness in individuals with RP. Successful implementation of this therapy requires addressing various critical factors, such as refining the targeting and expression of light-sensitive proteins, ensuring the sustained functionality of modified cells over the long term, and devising dependable and non-invasive methods for delivering light [134]. Furthermore, thorough investigations are needed to evaluate the therapy’s enduring effects, potential side effects, and its compatibility with individual patient profiles [135]. Nevertheless, optogenetic therapy presents a promising approach for treating RP and represents an exciting avenue for future research and clinical applications [136,137]. In the following section, we provide brief descriptions of a few optogenetic therapies currently under exploration for the treatment of retinitis pigmentosa:

- Channelrhodopsin-based therapy: Channelrhodopsin-2 (ChR2) is a light-sensitive protein derived from algae. In optogenetic therapy for RP, ChR2 is introduced into RGCs or bipolar cells. When activated by light of specific wavelengths, ChR2 can depolarize the cells and initiate electrical signals, mimicking the function of photoreceptor cells. This approach aims to restore light sensitivity and enable visual information to be transmitted to the brain [138].
- Halorhodopsin-based therapy: Halorhodopsin (NpHR) is a light-sensitive protein that responds to yellow or amber light. In optogenetic therapy, NpHR can be introduced into bipolar cells or RGCs to allow the cells to be inhibited in response to light stimulation. By selectively inhibiting specific cell types, such as ON or OFF bipolar cells, the retinal circuitry can be modulated to enhance visual processing and restore functional vision [139,140].
- Red-shifted opsin-based therapy: In addition to ChR2 and NpHR, other light-sensitive proteins with red-shifted absorption spectra are being explored for optogenetic therapy in RP. These proteins, such as ReaChR or ChrimsonR, can be activated by longer wavelengths of light, including red or near-infrared light. By utilizing these red-shifted opsins, optogenetic therapy can potentially penetrate deeper into the retina and improve light sensitivity in RP patients [141].

### 3.6. Strategies for Promoting the Survival and Function of Existing Retinal Cells

Another aspect of cell-based therapies for RP involves promoting the survival and function of existing retinal cells. Various approaches, including the transplantation of supportive cells or the delivery of neurotrophic factors, are being explored to enhance survival and protect the remaining retinal cells from further degeneration [142]. The neuroprotection and cell survival enhancement in RP could be achieved through the administration of neurotrophic factors like brain-derived neurotrophic factor (BDNF), ciliary neurotrophic factor (CNTF), and glial cell line-derived neurotrophic factor (GDNF) delivered to the retina with intravitreal injections where they promote the survival of retinal cells [143,144]. Besides neurotrophic factors, anti-apoptotic agents, such as caspase inhibitors and Bcl-2 family proteins, can be used to prevent or reduce the apoptosis of retinal cells and promote their survival [145]. As previously mentioned, apoptosis stands out as one of the key pathomolecular processes in RP, along with the persistent inflammatory response. Consequently, researchers are investigating anti-inflammatory strategies aimed at diminishing inflammation and its harmful impact on retinal cells [146]. This objective can be realized through the use of anti-inflammatory medications, immunomodulatory therapies, or other interventions targeting inflammatory pathways and cytokines. Lastly, a cutting-edge approach involves the management of oxidative stress by administering antioxidants and reactive oxygen species scavengers, such as vitamin C, vitamin E, and coenzyme Q10, in order to mitigate oxidative stress and safeguard retinal cells from damage [147,148].

## 4. Conclusions

Based on what has been discussed so far, it is clear that the comprehensive understanding of the pathogenetic mechanisms underlying retinitis pigmentosa represents the key to the development of new therapies and the enhancement of existing treatments. Since narrative reviews, by their very characteristics, usually lack rigorous methodology and inclusion and exclusion criteria as per the research articles, readers should be aware of such limitations when reading this wide-ranging view of new approaches to innovative therapies. Nevertheless, it is important to note that while cell-based therapies hold great potential, they are still in the experimental stages, and further research is needed to refine their safety and efficacy. The proof of concept that a new era of treating rare diseases is about to start is the increasing number of ongoing clinical trials that are assessing the feasibility and effectiveness of these approaches in treating RP. A close collaboration between researchers, clinicians, and regulatory bodies is warranted for their successful translation into clinical practice [149,150]. Relevant clinical trials with cell-based therapies are listed in Table 2.

## Figures and Tables

**Table 1:** Functions, effects of mutations, and effects on the retina’s structure of the major genes commonly involved in the pathogenesis of retinitis pigmentosa.

GENE/PROTEIN	FUNCTION	EFFECTS OF MUTATIONS	EFFECTS ON RETINA’S STRUCTURE
**Rhodopsin (*RHO*)**	Found in rod cells, plays a central role in phototransduction and rod photoreceptor cell health	Alteration of the protein’s structure/function, abnormalities in the protein’s folding or trafficking	Disruption and degeneration of rod photoreceptor cells, defects in phototransduction, reduced sensitivity to light
**Peripherin/RDS (*PRPH2*)**	Found in rod and cone cells, plays a crucial role in the structural integrity and organization of the photoreceptor outer segments (essential for disk morphogenesis)	Alteration of protein folding, stability, and interactions with other proteins	Alteration of integrity and function of the outer segments, progressive degeneration of the photoreceptor associated with peripheral vision loss, and central vision impairment
**Cyclic Nucleotide-Gated (*CNG*) Channels**	Nonselective cation channels located in the outer segment of rod and cone photoreceptor cells, involved in the regulation of ion influx in response to light stimulation	Impairs the normal function of these channels	Abnormalities in the phototransduction process, reduced sensitivity to light, decreased visual acuity, and progressive vision loss; dysfunctional CNG channels can lead to cellular stress, oxidative damage
**Retinal Pigment Epithelium-Specific 65 kDa Protein (*RPE65*)**	Component of the vitamin A visual cycle of the retina, which supplies the 11-cis retinal chromophore of the photoreceptors opsin visual pigments	Loss or dysfunction of the RPE65 protein, disrupting the visual cycle and impairing the regeneration of 11-cis-retinal	Progressive loss of photoreceptors reduced sensitivity to light, decreased visual acuity
**Retinitis Pigmentosa GTPase Regulator (*RPGR*)**	Predominantly localized in the connecting cilium and outer segment of photoreceptor cells in the retina, plays critical roles in the phototransduction cascade	Impairs the normal function	Impaired ciliary transport, altered protein–protein interactions, or disrupted signaling pathways, leading to photoreceptor cell death and vision loss
**Cone-Rod Homeobox Protein (*CRX*)**	Photoreceptor-specific transcription factor, which plays a role in the differentiation of photoreceptor cells; this homeodomain protein is necessary for the maintenance of normal cone and rod function	Disrupts the normal function	Impaired development and function of photoreceptor cells associated with degeneration of cones and rods
**Usher Syndrome Genes** **(*MYO7A, USH1C, USH2A, GPR98, CLRN1*)**	***MYO7A*** encodes the protein myosin VIIA, involved in the development and maintenance of photoreceptor cells	Impairs the normal function	Impaired development and function of photoreceptor cells associated with degeneration
	***USH1C*** encodes a scaffold protein involved in the organization of hair cell stereocilia and synaptic connections in the retina		
	***USH2A*** encodes a protein that contains laminin EGF motifs involved in the maintenance of the structure and function of photoreceptor cells (maintenance of periciliary membrane complex)		
	***GPR98*** encodes the protein ADGRV1 involved in the development of photoreceptors (maintenance of periciliary membrane complex)		
	***CLRN1*** encodes the protein Clarin 1, which plays an important role in the development and homeostasis of photoreceptor cells (regulatory element for the synapses within the retina)		

**Table 2:** Relevant clinical trials with cell-based therapies for the treatment of retinitis pigmentosa www.clinicaltrials.gov (accessed 2 August 2023), last updated 2 August 2023.

ID	NAME	PHASE	AIM	METHODS
NCT02320812	*A Prospective, Multicenter, Open-Label, Single-Arm Study of the Safety and Tolerability of a Single, Intravitreal Injection of Human Retinal Progenitor Cells (jCell) in Adult Subjects With Retinitis Pigmentosa (RP)*	1/2	Test the safety, tolerability, and efficacy (impact on visual status) of the administration of a single dose of jCell	Single intravitreal injection of 0.5–3.0 × 10^6^ human retinal progenitor cells (hRPC-jCell)
NCT04925687	*Phase 1 Study of Intravitreal Autologous CD34^+^ Stem Cell Therapy for Retinitis Pigmentosa (BMSCRP1)*	1	Determine the safety and feasibility of injection of autologous CD34^+^ stem cells harvested from bone marrow	Intravitreal injection of autologous CD34^+^ cells harvested from bone marrow under GMP conditions
NCT04763369	*Investigation of Therapeutic Efficacy and Safety of UMSCs for the Management of Retinitis Pigmentosa (RP)*	1/2	Investigate the safety and therapeutic efficacy of umbilical cord-derived mesenchymal stem cell (UC-MSC) injection, employing two different routes (sub-tenon injection versus suprachoroidal injection)	Sub-tenon and suprachoroidal injection of UC-MSCs
NCT05909488	*Role of UC-MSC and CM to Inhibit Vision Loss in Retinitis Pigmentosa Phase I/II*	2/3	Investigate the safety and therapeutic efficacy of peribulbar injection of umbilical cord-derived mesenchymal stem cell (UC-MSC) with conditioned medium (CM)	Peribulbar injection of 1.5–5 × 10^6^ UC-MSC + CM
NCT03944239	*Safety and Efficacy of Subretinal Transplantation of Clinical Human Embryonic Stem Cell-Derived Retinal Pigment Epitheliums in Treatment of Retinitis Pigmentosa*	1/2	Test the safety and therapeutic efficacy of clinical-level human embryonic stem cell-derived retinal pigment epithelium transplantation	Subretinal transplantation of clinical human embryonic stem cell-derived retinal pigment epitheliums
NCT01531348	*Intravitreal Injection of MSCs in Retinitis Pigmentosa*	1	Determine feasibility and safety of adult human bone marrow-derived mesenchymal stem cells (BM-MSC) by intravitreal injection	Intravitreal injection of 1 × 10^6^ BM-MSC in a balanced salt solution
NCT03073733	*Safety and Efficacy of Intravitreal Injection of Human Retinal Progenitor Cells in Adults With Retinitis Pigmentosa*	2	Evaluation of safety and efficacy of intravitreal injection of human retinal progenitor (hRPC)	Intravitreal injection of 3.0–6.0 × 10^6^ of human retinal progenitor cells (hRPC) suspended in clinical-grade medium
NCT04284293	*CNS10-NPC for the Treatment of RP*	1	Assess the safety and tolerability of two escalating doses of clinical-grade human fetal cortical-derived neural progenitor cells (CNS10-NPC); determine if CNS10-NPC can engraft and survive long-term in the retina of transiently immunosuppressed subjects; obtain evidence that subretinal injection of CNS10-NPC can favorably impact the progression of vision loss in subjects with moderate RP	Human neural progenitor cell (CNS10-NPC) sub-retinal space implantation
NCT02709876	*Autologous Bone Marrow-Derived CD34^+^, CD133^+^, and CD271^+^ Stem Cell Transplantation for Retinitis Pigmentosa*	1/2	Assess the safety and efficacy of purified adult autologous bone marrow-derived CD34^+^, CD133^+^, and CD271^+^ stem cells through a 48-month follow-up period. The combination of these three cell types was based on their diverse potentialities to differentiate into specific functional cell types to regenerate damaged retinal tissue	Intravitreal injection of bone marrow-derived CD34^+^, CD133^+^, CD271^+^ stem cells in 1.0 mL normal saline
NCT03963154	*Interventional Study of Implantation of hESC-derived RPE in Patients With RP Due to Monogenic Mutation*	1/2	Study the safety, tolerability, and preliminary efficacy of implantation into one eye of human embryonic stem cell-derived retinal pigment epithelium (hESC-derived RPE))	Implantation into one eye of human embryonic stem cell-derived retinal pigment epithelium (hESC-derived RPE)
NCT03566147	*Treatment of RP and LCA by Primary RPE Transplantation*	Early 1	Study the safety and preliminary efficacy of human primary retinal pigment epithelial (HuRPE) cells subretinal transplantation	Subretinal space transplantation of 0.3–1 × 10^6^ HuRPE cells through a standard surgical approach
NCT03772938	*Stem Cells Therapy in Degenerative Diseases of the Retina*	1	Investigation of the safety and efficacy of intravitreal injection of autologous bone marrow-isolated stem/progenitor cells with different selected phenotypes; this clinical trial was specially designed to test the therapeutic (pro-regenerative and neuro-protective) functions of different stem/progenitor cell populations able to secrete bioactive neurotrophic factors	Intravitreal injection of human autologous bone marrow-derived stem/progenitor cell
NCT05147701	*Safety of Cultured Allogeneic Adult Umbilical Cord-Derived Mesenchymal Stem Cells for Eye Diseases*	1	Study the safety and efficacy of intravenous and sub-tenon delivery of cultured allogeneic adult umbilical cord-derived mesenchymal stem cells (UC-MSCs)	Intravenous and sub-tenon injection of 1 × 10^6^ allogeneic adult umbilical cord-derived mesenchymal stem cells

## Data Availability

The authors confirm that the data supporting the findings of this study are available within the article.

## Abbreviations

RP	Retinitis pigmentosa
RPE	Retinal pigment epithelium
CME	Cystoid macular edema
adRP	Autosomal dominant retinitis pigmentosa
NpHR	Halorhodopsin
BDNF	Brain-derived neurotrophic factor
ChR2	Channelrhodopsin-2
GDNF	Glial cell line-derived neurotrophic factor
GFAP	Glial fibrillary acidic protein
ESCs	Embryonic stem cells
iPSCs	Induced pluripotent stem cells
ROS	Reactive oxygen species
OECs	Olfactory ensheathing cells
MSCs	Mesenchymal stromal cells
RGCs	Retinal ganglion cells
CNTF	Ciliary neurotrophic factor
